# The writing on the wall

**DOI:** 10.7554/eLife.00642

**Published:** 2013-03-26

**Authors:** Henry R Bourne

**Affiliations:** Department of Cellular and Molecular Pharmacology, University of California at San Francisco, United States. He blogs about the challenges and opportunities associated with biomedical research at biomedwatch.wordpress.com. His research was supported by the NIH from 1969 to 2008, and he served as a reviewer of grant applications at intervals during this period. henry.bourne@ucsf.edu

**Keywords:** point of view, science policy, careers in science, NIH, postdoc, grad school, funding

## Abstract

The biomedical research enterprise in the US has become unsustainable and urgent action is needed to address a variety of problems, including a lack of innovation, an over-reliance on soft money for faculty salaries, the use of graduate students as a source of cheap labour, and a ‘holding tank’ full of talented postdocs with limited career opportunities.

Belshazzar, the last King of Babylon, is famous for holding a banquet at which a disembodied hand wrote four words on the wall of his palace. Unable to understand what the words meant, he called for the prophet Daniel, who told him that the Babylonian kingdom was coming to an end. That night the Persian army entered the city of Babylon and Belshazzar was killed.

Millennia later, biomedical scientists and research institutions in the US appear equally unable to read the writing on the wall. Obsessed by threats to federal funding, they misread clear warnings on every wall: without major change, the US biomedical research enterprise cannot be sustained; labs will shrink; a generation of young scientists will be lost; competition will stymie innovation; and young scientists and poor institutions will lose out as senior investigators and rich research centres grow richer.

**Figure 1:**
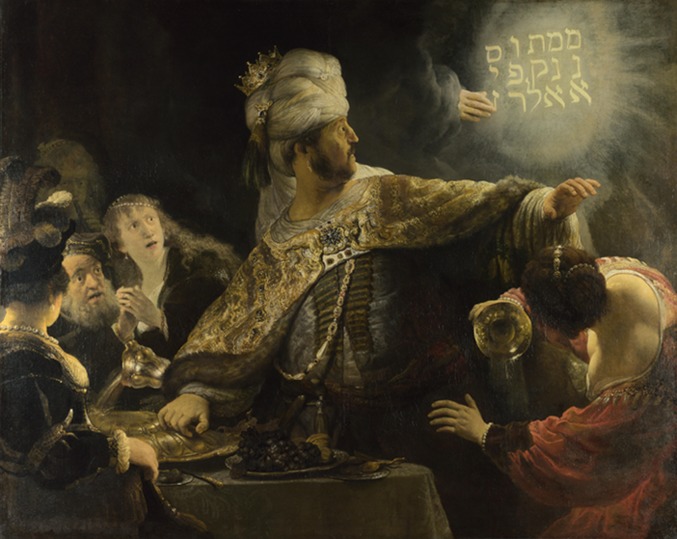
Belshazzar, the last King of Babylon, depicted here in a painting by Rembrandt, was killed after he failed to read the writing on the wall. More than two millennia later, the biomedical research enterprise in the US finds itself in a similar position: addicted to expansion after four decades of increasing budgets, it needs to rethink how it funds research and researchers to ensure that it has a sustainable future.

IMAGE: REMBRANDT [PUBLIC DOMAIN], VIA WIKIMEDIA COMMONS.

The inexorable decline of the immense biomedical research enterprise in the US may serve merely to show other countries how not to manage biomedical research. Alternatively, we may hope that the stakeholders in biomedical research in the US will wake up, read the writing on the wall, and act. Congress, state governments, the National Institutes of Health (NIH), the National Science Foundation, the foundations that support research, the principal investigators on research grants, and the research institutions themselves (that is, universities, medical schools and research institutes) all helped create our present predicament. While solving it requires all to contribute, I focus here on three crucial stakeholders: principal investigators and institutions, both of whom try to ignore the sustainability problem, and the NIH, which tiptoes gingerly around it.

## The roots of the problem

Even the principal investigators and the institutions must now suspect that, despite their protests and fervent pleas for more money, funding for biomedical research in the US is going to contract significantly over the near term, and probably longer. They blame their present troubles—specifically, almost a decade of flat-lined NIH budgets—on Congressional gridlock and a weak economy when, in fact, they should share responsibility for creating an even more serious problem as a result of their actions and decisions over the past four decades. The annual budget of the NIH increased significantly over this period, rising from $1.06 billion in 1970 to $28.6 billion in 2005: annual growth averaged about 9% between 1970 and 1998, and was close to 15% during the subsequent five ‘doubling’ years (Korn et al., 2002). Well before the century turned, therefore, the biomedical research enterprise in the US had become addicted to expansion. Competition-driven feedback loops led institutions to hire more investigators, build more labs, make faculty salaries more dependent on NIH dollars (‘soft’ money), and attract more trainees to do the work—thus driving even fiercer competition among institutions and scientists.

The idea that excessive competition can block progress appears counterintuitive to many, but analysis of biomedical research funding shows this is the case (Teitelbaum, 2008; Stephan, 2011). Competition drives scientific discovery, but too much competition for scarce resources can block progress, and has done so. Thus, the growing flood of grant applications surpasses growth in NIH dollars, reduces the proportion of grants that are funded, and renders peer review increasingly arbitrary because a project ranked in the 20th percentile is often no less meritorious than one ranked in the 10th percentile (Berg, 2013).

Another problem is that we now have a ‘holding tank' of postdoctoral scholars that is overflowing with bright young scientists who are indentured to greying lab chiefs and are thus unable to break new ground as independent researchers (Bourne, 2012). The worst consequence, but harder to quantify, is that scientists avoid risky, creative projects in favour of ‘sure things’ more likely to be funded by conservative reviewers (Nicholson and Ioannidis, 2012).

The NIH appointed a Biomedical Workforce Task Force to propose how to deal with some of these problems. Although the task force appeared to recognize that the biomedical research enterprise in the US is not presently sustainable, its recommendations and the NIH's actions (so far) failed to grapple with the excessive dependence of research faculty salaries on grants, the need to separate the funding of graduate trainees from the funding of research, or the postdoc holding tank (Biomedical Workforce Task Force, 2012).

## Six questions and my answers to them

So what do the stakeholders in biomedical research in the US need to do? In my opinion they must unite to craft strategies that nourish the best young scientists, to prune less creative projects, and to make biomedical research able to sustain itself. Achieving this will require the stakeholders to answer a number of difficult questions. Below I list six of these questions, along with my answers to them.

### How can we reduce the growing reliance on soft-money PI salaries?

Medical schools and research institutes routinely rely on NIH research project grants to pay large proportions of PI salaries—a practice that is also beginning to afflict research universities. Soft money salaries weaken collegial bonds among scholars, make researchers less willing to teach, undermine loyalties of faculty and institutions to one another, and discourage innovation by PIs. The faintest whisper of curtailing soft-money salaries strikes terror into financially threatened academic institutions, but they would be well advised to put more of their own skin into the research game. Institutions should join PIs and the NIH to schedule gradual but substantial change. At first, perhaps, no faculty investigator should receive more than 90% of their salary from research project grants; if the bar were raised 10% every five years thereafter, within 20 years no PI would receive more than 50% of their salary from grants.

Soft money salaries weaken collegial bonds among scholars, make researchers less willing to teach, undermine loyalties of faculty and institutions to one another, and discourage innovation by PIs.

### How can we revise federal ‘indirect cost’ rules that reward behaviour that is not sustainable?

Innovative research depends more on good ideas, which generally come from PIs, than on laboratory bricks and mortar. However, the rules on reclaiming indirect costs on grants from the NIH and other federal agencies reward institutions for diverting funds that could be used to pay PI salaries into building new laboratories instead. The diversion reflects two problems (Alberts, 2010): (i) when an institution uses its own money to pay the salary of a PI, this is not counted as a ‘direct’ cost of research, so the institution can claim less for indirect costs from the NIH than they can when the salary of the PI is paid from the grant; (ii) institutions can reclaim certain indirect costs related to the construction of new buildings and laboratories. Until these rules are changed, research institutions would be crazy not to invest scarce funds in buildings rather than people.

### How can we stop paying PhD trainees from research project grants?

Institutions and PIs are recruiting more and more young PhD trainees as cheap labour for their labs, and paying their stipends from research project grants rather than training grants. This has three adverse consequences: first, floods of newly minted PhDs apply for postdoctoral positions and jobs in industry, in numbers that drive down their salaries in whatever job they can get; second, institutions track trainees paid from training grants, but they do a terrible job of tracking the very large number of trainees paid from research project grants, making it impossible to know their numbers and eventual fates (knowledge that is essential for regulating the numbers of PhDs and postdocs); third, although trained to a very high level in laboratory research, too many PhD graduates settle for jobs that do not involve research and may not even relate to science, thereby wasting trainee positions and research dollars (Biomedical Workforce Task Force, 2012). The NIH focuses on the third of these issues, but it appears to ignore the obvious remedy for all three: to support all PhD trainees on training grants, not research project grants. Making this change in a gradual manner would be possible, but not easy, if PIs and institutions cooperated with the NIH.

### How can we create a sustainable laboratory workforce?

The dangers of excessive competition among PIs and institutions, as described above, will intensify as long as training for young scientists (both graduate students and postdocs) is driven by the demand for cheap lab workers, rather than by sustainable increases in positions for new PIs. Thus it is crucial for labs to hire more staff scientists and fewer junior faculty supported primarily on grants, while maintaining a reduced but still substantial number of postdocs and young scientists in training positions. To promote such changes in laboratory personnel, PIs and institutions must work with the NIH to define both the roles and support mechanisms for staff scientists.

### How can we shrink the postdoc ‘holding-tank’?

This goal will also require concerted efforts of both institutes and the NIH to shorten postdoctoral service to a maximum of five years, to eliminate quasi-faculty slots that lack all institutional salary support, and to impose more stringent quality controls on entry of both US and foreign PhDs into the ‘tank’.

### How can we ensure the appropriate balance between investigator-driven research into fundamental biological mechanisms, ‘big science’ and translational projects?

The size and complexity of the NIH make it difficult to even identify and quantify how much is spent on these three areas, and it is even harder to compare them in terms of ‘bangs for your buck’. There is a vast literature on this topic but, so far as I can see, very little meaningful data. Along with many colleagues, however, I strongly suspect that current trends at NIH dangerously limit its investment in research aimed at explaining fundamental biological mechanisms. All stakeholders in biomedical research must work together to resolve these issues, relying less on pontification (by me and everyone else, whatever their opinions) and more on data and critical analysis.

## Cooperation is required

Why do universities and PIs remain silent on questions that affect sustainability of their cherished research enterprise? Rather than sheer stupidity, I suspect the answer is blind arrogance: they fondly imagine that more vulnerable competitors will succumb to the current financial scourge, allowing the stronger, brighter dinosaurs (that is, themselves) to slither from their bunkers and garner whatever resources remain. Bunkers may suffice for a while, but even a thunder lizard can't survive long on magic alone. Instead, the changes needed to sustain biomedical research require hard thinking and cooperation among all biomedical research stakeholders. The NIH cannot solve the problems by itself. Researchers should argue strongly for sustained federal support, but it is delusional to expect a return to the era of regular 9% annual funding increases. We need no prophets to read the writing on the wall. Instead, for the foreseeable future, we must all sustain the enterprise with what we can get.
